# Clinicopathologic Significance and Prognostic Value of Programmed Cell Death Ligand 1 (PD-L1) in Patients With Hepatocellular Carcinoma: A Meta-Analysis

**DOI:** 10.3389/fimmu.2018.02077

**Published:** 2018-09-11

**Authors:** Jing-Hua Li, Wei-Jie Ma, Gang-Gang Wang, Xiang Jiang, Xi Chen, Long Wu, Zhi-Su Liu, Xian-Tao Zeng, Fu-Ling Zhou, Yu-Feng Yuan

**Affiliations:** ^1^Department of Hepatobiliary and Pancreatic Surgery, Zhongnan Hospital of Wuhan University, Wuhan, China; ^2^Department of Evidence-Based Medicine and Clinical Epidemiology, Center for Evidence-Based and Translational Medicine, Second Clinical College of Wuhan University, Zhongnan Hospital of Wuhan University, Wuhan, China; ^3^Department of Hematology, Zhongnan Hospital, Wuhan University, Wuhan, China

**Keywords:** programmed cell death ligand-1, hepatocellular carcinoma, prognosis, clinicopathology, meta-analysis

## Abstract

**Background:** There is still a dispute over an issue of the clinical pathology and prognostic of programmed cell death ligand 1 (PD-L1) in hepatocellular carcinoma (HCC) patients. Here, we undertook this meta-analysis to survey the conceivable role of PD-L1 in HCC.

**Method:** We searched databases like MEDLINE, EMBASE, and Google Scholar for relevant studies published in English up to February 13, 2018. We implemented the appraisal of the eligible studies according to the choice criterion. We used Hazard ratio (HR) and its 95% confidence interval (95% CI) to evaluate the prognostic role of PD-L1 for overall survival (OS), disease-free survival (DFS), and recurrence-free survival (RFS). Odds ratio (OR) and the corresponding 95% CI were calculated to evaluate the connection between PD-L1 and clinicopathological features. Publication bias was tested.

**Results:** 13 studies, which published range from 2009 to 2017 were contained in this meta-analysis, involving 1,843 patients with HCC. The results indicated that high PD-L1 could predict shorter OS (HR = 1.57, 95% CI: 1.09-2.27, *P* < 0.00001) as well as poorer DFS (HR = 2.07, 95% CI: 1.20-3.58, *P* = 0.009). Additionally, high PD-L1 expression was correlated to liver cirrhosis (OR = 1.66, 95% CI: 1.10-2.50, *P* = 0.02), poorer tumor Barcelona Clinical Liver Cancer (BCLC) stage (OR = 0.30, 95% CI: 0.10-0.88, *P* = 0.03) and portal vein invasion (OR = 1.96, 95% CI: 1.04-3.68, *P* = 0.04), but had no correlation with age, gender, tumor size, number of tumors, AFP, vascular invasion, HBVs-Ag, Anti-HCV, differentiation or TNM stage. Besides, no significant publication bias was found among these identified studies.

**Conclusion:** The meta-analysis suggested that PD-L1 overexpression could foresee worse OS and DFS in HCC. Moreover, the PD-L1 expression has to bear on liver cirrhosis, portal vein invasion, and BCLC stage.

## Introduction

Hepatocellular carcinoma (HCC) is the third most frequent cause of cancer death worldwide (1). People with early-stage HCC may benefit from potentially curative therapies. Unfortunately, a significant proportion of patients is diagnosed at a middle or advanced stage. Therefore, the prognosis is inferior for these patients, and the effective treatment options are limited (2). Recently, many studies find that PD-L1, implicated in CD8 T-cell tolerance, is overexpressed in various solid tumor cells, such as HCC (3–7), melanoma (8, 9), colorectal cancer (10), lung cancer (11–14) and pancreatic carcinoma (15). Some articles also indicate that the survival and tumor recurrence of HCC patients are affected by the PD-L1 overexpression. PD-L1 overexpression may reflect the presence of endogenous host immune response to tumor and serve as a biomarker for predicting survival benefits from adjuvant CIK cell immunotherapy in HCC patients (16). And peritumoral PD-L1 expression was associated with significantly worse survival compared to the negative expression group (17). However, the results of some studies are reversed. The research data showed that lack of, or low, tumor expression of PD-L1 was associated with poor HCC-specific survival (18). Therefore, there is a need to combine the conflicting data to have an explicit clarification.

PD-L1, also known as B7-H1 or CD274, is an essential member of the B7/CD28 costimulatory factor superfamily (19).PD-L1 expressed on cancer cells can inhibit T-cell activation, maintain the exhaustion of T cell, impairing cytokine production, and induce the apoptosis of effector T cells which is attributable to the growth of the tumor (20, 21). The combination of programmed cell death 1 (PD-1) and PD-L1 could attenuate IL-2 secretion and T-cell proliferation which were mediated by T cell receptor (22), explaining that overexpression of PD-L1 on potentially immunogenic tumor cells (23). And PD-L1 binds to the PD-1 receptor leading to the negative regulation of immune activity. Herein, the existing evidence demonstrates that the making sorafenib and anti-PD-L1 monoclonal antibody (mAb) combined to treat can be applied to evaluate therapeutic efficacy for HCC (24, 25). Immune blocking therapy is a promising method for the treatment of malignant cancers. The monoclonal antibodies which target PD-1 and PD-L1 have incentive function and prolong the stabilization of diseases including various cancers, such as breast cancer and gastric cancer (26–28).

Moreover, previous studies have pointed out that the expression level of PD-L1 in HCC patients could serve as a predictive biomarker for the cytokine-induced killer (CIK) cell immunotherapy (16). Until now, Food and Drug Administration (FDA) has approved several PD-L1-targeted immune checkpoint inhibitors such as Atezolizumab, Avelumab, and Durvalumab. Recently, clinical trial research about the treatment of liver cancer, which made anti-PD-L1 antibody durvalumab and the anti-CTLA-4 antibody combined showed that response rates were inspiring (29). However, PD-1 and PD-L1 as therapeutic targets for HCC are still demanded, which need further evaluation (30).

Therefore, in this work, we performed this meta-analysis to assess the prognosis value of PD-L1 in HCC, and to highlight the development of PD-L1/PD-1 immune checkpoint targeted therapy.

## Method

### Eligibility criteria

The eligible records which used random controlled trials must meet the inclusion criterions as follows: (1) articles were published in English with full-texts, using human beings as study subjects; (2) every patient was diagnosed as hepatocellular carcinoma; (3) PD-L1 expression levels were gauged in clinical HCC tissues; (4) the correlation between PD-L1 and survivals (OS/RFS/DFS) was detected; (5) the relation of PD-L1 with clinicopathological features was assessed based on no fewer than two parameters; (6) researches supplied abundant materials to calculate hazard ratios (HR), odds ratio (OR) and their 95% confidence interval (95% CI). Studies that had insufficient data were excluded. We chose to analyze the latest or most comprehensive data when we disposed of repetitive studies.

### Literature search strategy

We searched the online databases of MEDLINE, EMBASE, and Google Scholar for interrelated researches reported in English up to February 13, 2018, using the keywords as below: “hepatocellular carcinoma or hepatocellular cancer or HCC or liver cancer” and “programmed cell death-ligand 1 or PD-L1 or B7-H1.”

### Data extraction

Two reviewers extracted the available data independently (Li JH and Ma WJ). Any disagreements over the information were resolved by consensus or by the judgment of a third reviewer (Wang GG). The name of first author, year of publication, country, the method for detection of PD-L1, cut-off value, follow-up time, clinicopathological parameters and HRs with 95% CIs were derived for survival analyses. HRs were extracted from two methods as follows: (1) HRs and 95% CIs were acquired immediately from the articles; (2) We obtained HRs and 95% CIs from the Kaplan-Meier survival curves through using Engauge Digitizer version 4.1. The second way may cause errors due to variation. Besides, ORs and 95% CIs were adopted to investigate the connection between the level of PD-L1 and clinicopathologic characteristics.

### Quality assessment

Two authors (Jiang X and Chen X) independently evaluated the quality of all literature in the light of a 9-score system of the Newcastle-Ottawa Scale (NOS). We settled the discrepancies in the score through discussion and analysis among the authors. Judging every study was on the grounds of three aspects: (I) the selection of the groups of study (four items, one score each); (II) the comparability (one item, up to two scores); and (III) the ascertainment of either the exposure or outcome of interest (three items, one score each). A score on behalf of a high-quality choice of individual study.

### Statistical analysis

The Cochrane Q-test and *P*-values were used to assess the heterogeneity between studies. Statistically significant heterogeneity was defined as a chi-squared *P*-value < 0.1 or an *I*^2^ statistic > 50%. Then the random effects model would be performed if inter-study heterogeneity was significant; or else, the fixed effects model was utilized.

We used HRs with their 95% CIs to measure the value of PD-L1 in the survival for HCC. HR > 1 meant a poorer survival for the higher PD-L1 expression group. In contrast, HR < 1 represented a worse survival for the lower PD-L1 expression group. ORs and 95% CIs were computed to assess the relationship between PD-L1 over-expression and clinicopathologic features. OR > 1 meant a poorer prognosis for the higher expressed PD-L1 group. Inversely, OR < 1 implied a worse prognosis for the decreased PD-L1 expression group. We extracted relevant data from the identified studies, and ORs and HRs were estimated by a meta-analysis.

All data syntheses in this meta-analysis were performed using the Revman5.3 Software (Revman, the Cochrane Collaboration) and the Stata11.0 Software (Stata, College Station). Sensitivity analysis was conducted by sequentially omitting each study one at a time to inspect the stability of our estimates. The potential publication bias was estimated by Begg's funnel plot and Egger's test, and *P* < 0.05 implied statistical significance.

## Results

### Search results

**Figure 1:**
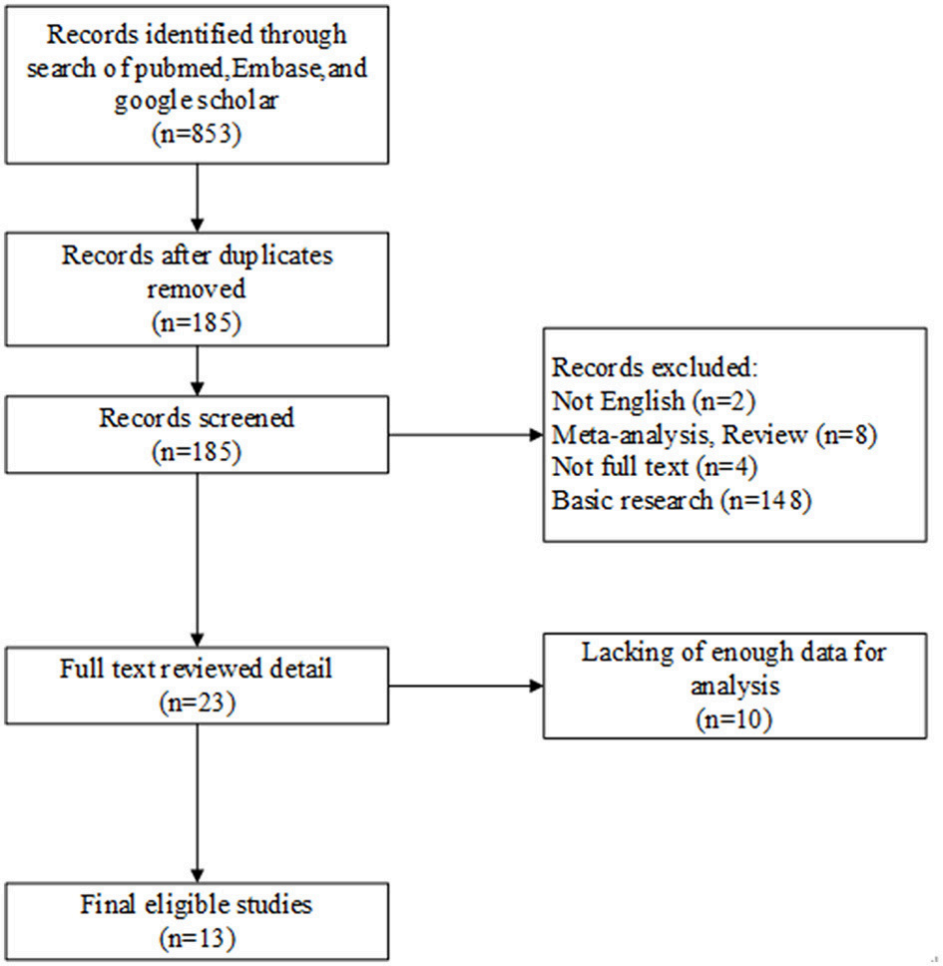
Flow chart of studies in the analysis.

We searched several international databases, and there were a total of 853 articles were incipiently included. Of these, 668 duplicates were excluded, and we deleted another 162 records after screening titles or abstracts for non-English papers, meta-analysis, reviews, no full-texts, and irrelevancy. Next, another 10 reviews were further removed from the remaining 23 records due to insufficient data. Hence, the article contained a total of 13 studies. Figure 1 demonstrates the particular selection process.

### Study characteristics

Table 1 listed the principal features of the included studies. The scope of the publication year was from 2009 to 2017. The total number of patients in all studies was 1,843, has a range of 58–240. Nine studies were implemented in Asian countries, and four were reported in western countries. Four articles did not report HRs and 95% CIs directly, so we calculated these statistics by adopting the Kaplan-Meier curves.

**Table 1:** The characteristics of studies included in the meta-analysis.

**References**	**Country**	**Method**	**Outcome**	**Case number (High/Low)**	**Cut-off**	**Follow up time**
Dai et al. (17)	China	Tissue Microarray, Immunohistochemistry	OS DFS	90(28/62)	Staining scores <2	Median time 60.8 month
Sideras et al. (18)	Netherlands	Tissue Microarray, Immunohistochemistry, PCR	RFS	EMC86(70/16)	2 log likelihood	>100 months
				AMC 60(51/9)		
Semaan et al. (31)	Germany	Tissue microarray, Immunohistochemistry	OS	176(88/88)	Median	60 months
Xie et al. (32)	China	Immunohistochemistry	OS DFS	167(24/143)	Membranous staining = 5% of tumor cells	150 months
Chen et al. (16)	China	Immunohistochemistry	OS RFS	231(58/173)	Membranous staining percentage = 5%	>5 years
Jung et al. (30)	Korea	Immunohistochemistry	OS DFS	85(23/62)	Staining scores = 3	125 months
Umemoto et al. (33)	Japan	Immunohistochemistry	OS RFS	80(37/43)	Staining score = 1	median time 2,427 days
Zeng et al. (34)	China	Immunohistochemistry	OS DFS	156(88/68)	Staining score = 1	40 months
Gao et al. (6)	China	Tissue microarray, Immunohistochemistry	OS RFS DFS	240(60/180)	The 75th percentile	120 months
Gabrielson et al. (35)	America	Immunohistochemistry, semi-quantitative analysis	OS RFS	58(19/39)	NA	meantime 39.7 months
Kan et al. (36)	China	Immunohistochemical Staining	OS	128(105/23)	1 point	median time 10 months
Wu et al. (4)	China	Immunohistochemistry	OS	71(35/36)	The median value of B7-H1+cell density in tumor tissues on IHC	80 months
Finkelmeier et al. (37)	Germany	ELISA	OS	215(64/151)	0.8 ng/ml	298 ± 304 days with a range of 1–1464 days

### Survival analysis

**Figure 2:**
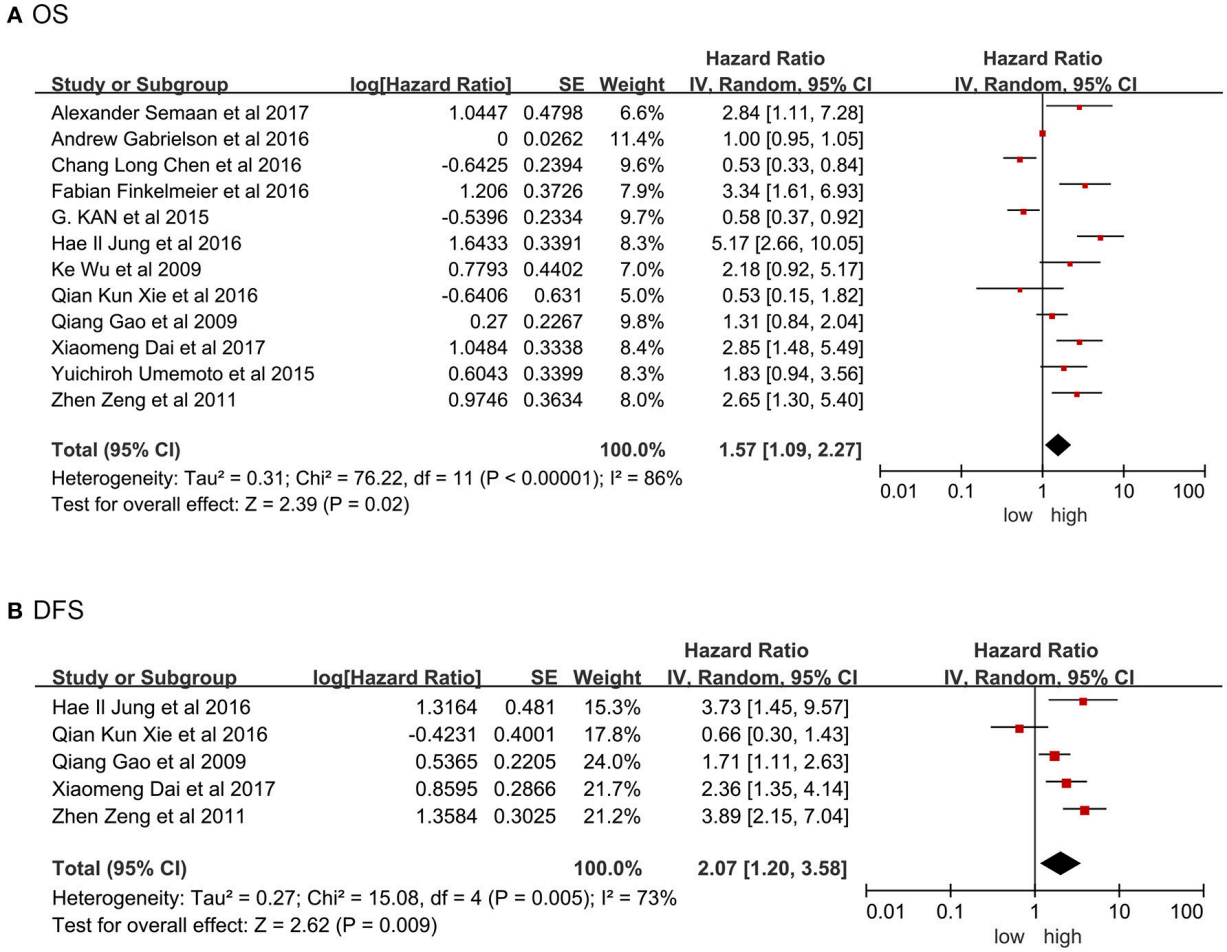
Forrest plot of HR for OS **(A)**, DFS **(B)**. Size of the square indicates the relative contribution of each study. The solid horizontal line represents 95% confidence interval of each study. The diamond indicates pooled studies.

Twelve studies were providing the data on overall survival (OS). Significant heterogeneity existed among included studies (Tau2 = 0.31; Chi^2^ = 76.22; *P* < 0.00001; *I*^2^ = 86%). Pooled results by random model revealed high PD-L1 was related to poor prognosis in term of shorter OS (HR = 1.57, 95% CI: 1.09-2.27, *P* = 0.02; Figure 2A). Figure 2B showed significant heterogeneity existed in the included studies involving disease-free survival (DFS; Tau2 = 0.27; Chi^2^ = 15.08; *P* = 0.005; *I*^2^ = 73%). Pooled results by random model uncovered that high PD-L1 predicted poorer DFS (HR = 2.07, 95% CI: 1.20-3.58, *P* = 0.009). In this meta-analysis, HR and 95% CI from five studies with recurrence-free survival (RFS) data demonstrated no significant connection between high PD-L1 and RFS (HR = 1.24, 95% CI: 0.76-2.00, *P* = 0.39), although overt heterogeneity existed among these studies as well (Tau2 = 0.24; Chi^2^ = 29.18; *P* < 0.00001; *I*^2^ = 86%; Figure S1a).

### Correlation of PD-L1 expression with clinical features

**Figure 3:**
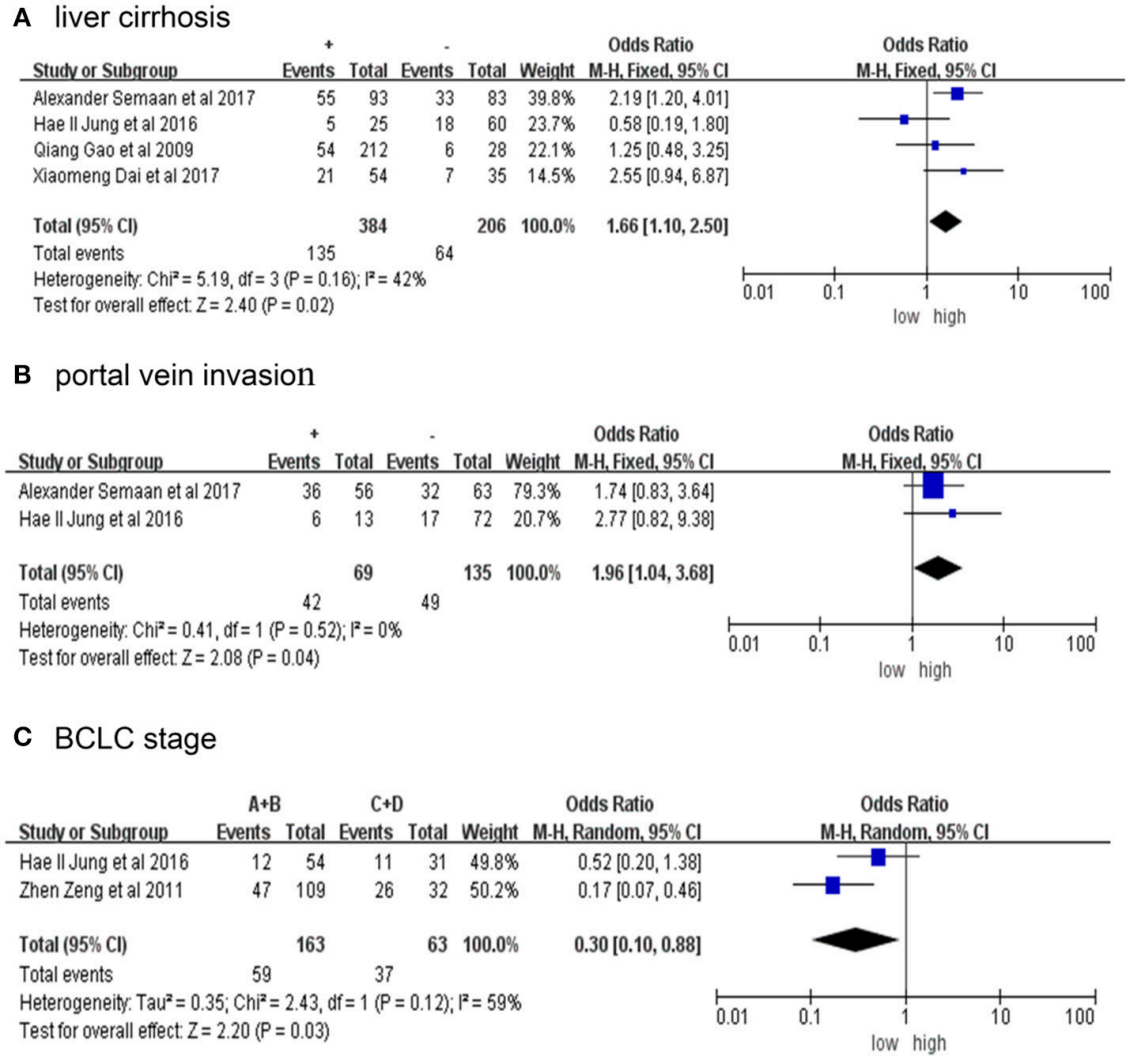
Forrest plot of HR for liver cirrhosis **(A)**, portal vein invasion **(B)**, BCLC stage **(C)** + represent positive, - represent negative. Size of the square indicates the relative contribution of each study. The solid horizontal line represents 95% confidence interval of each study. The diamond indicates pooled studies.

Table 2 presents the relationship between elevated PD-L1 and clinical parameters. High PD-L1 was found to be obviously correlated with positive liver cirrhosis (OR = 1.66, 95% CI: 1.10-2.50, *P* = 0.02; Figure 3A), positive portal invasion (OR = 1.96, 95% CI: 1.04-3.68, *P* = 0.04; Figure 3B) and poorer tumor Barcelona Clinical Liver Cancer (BCLC) stage (OR = 0.30, 95% CI: 0.10-0.88, *P* = 0.03; Figure 3C). Nevertheless, the PD-L1 expression has no obvious connection with age, sex, AFP, tumor size, number of tumors, vascular invasion, HBVs-Ag, Anti-HCV, differentiation or TNM stage (Figure S2).

**Table 2:** The relationship between high PD-L1 and the clinicopathological features.

**Characteristics**	**Studies**	**Case number**	**Pooled OR (95% CI)**	**P**	**Heterogeneity**		**Model**	**Publication bias begg's P**	**References**
					**I^2^**	**P**			
Age(<50 VS ≥50)	3	358	0.84 [0.49, 1.43]	0.52	0%	0.57	Fixed	1	(17, 32, 36)
Sex	8	1107	1.23 [0.85, 1.79]	0.27	0%	0.46	Fixed	0.386	(6, 17, 30–34, 36)
Tumor size(<5 cm VS ≥ 5 cm)	4	542	0.75 [0.50, 1.12]	0.12	49%	0.16	Fixed	1	(6, 17, 30, 36)
AFP(<20 ng/ml VS ≥20)	2	381	0.68 [0.42, 1.08]	0.41	0%	0.1	Fixed	1	(6, 34)
Liver cirrhosis	4	590	1.66 [1.10, 2.50]	0.02	42%	0.16	Fixed	0.308	(6, 17, 30, 31)
Number of tumors	5	726	0.84 [0.59, 1.20]	0.34	5%	0.38	Fixed	0.806	(6, 17, 31, 33, 34)
HBs-Ag	2	256	0.66 [0.18, 2.45]	0.54	69%	0.07	Random	1	(31, 33)
Anti-HCV	2	256	1.59 [0.86, 2.92]	0.14	14%	0.28	Fixed	1	(31, 33)
Portal vein invasion	2	204	1.96 [1.04, 3.68]	0.04	0%	0.52	Fixed	1	(30, 31)
Vascular invasion	4	556	1.92 [0.80, 4.60]	0.14	76%	0.005	Random	1	(6, 17, 30, 34)
BCLC stage(A+B VS C+D)	2	226	0.30 [0.10, 0.88]	0.03	59%	0.12	Random	1	(30, 34)
Differentiation(1 + 2 VS 3 + 4)	4	628	1.12 [0.43, 2.90]	0.81	73%	0.01	Random	0.308	(6, 30–32)
TNM stage (1+2 VS 3 + 4)	4	528	1.00 [0.65, 1.53]	0.99	0%	0.48	Fixed	0.174	(6, 32, 33, 36)

### Publication bias and sensitivity analyses

**Figure 4:**
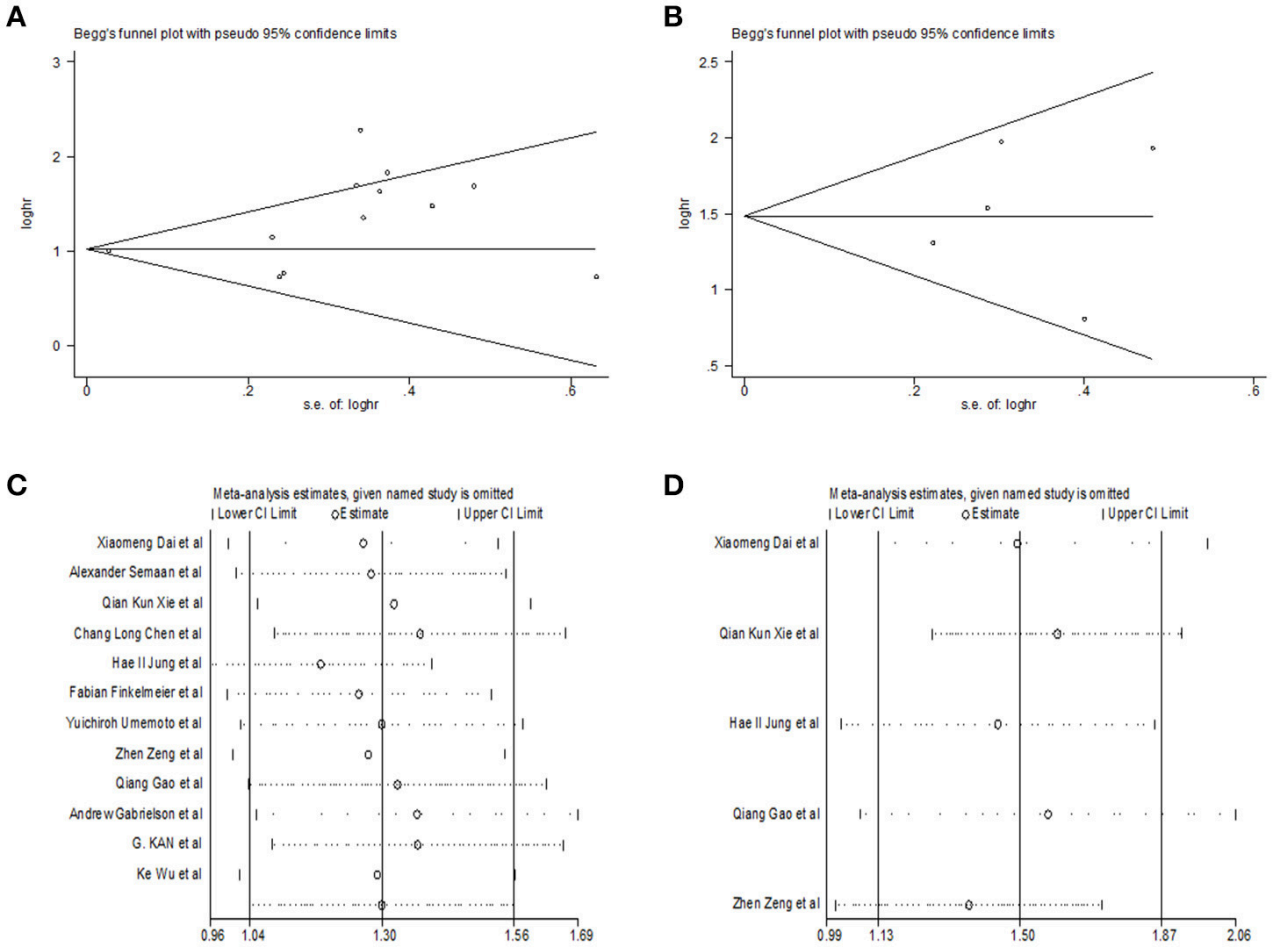
Begg's funnel plot for publication bias tests in **(A)** OS, **(B)** DFS. Sensitivity analysis in **(C)** OS, **(D)** DFS.

**Figure 5:**
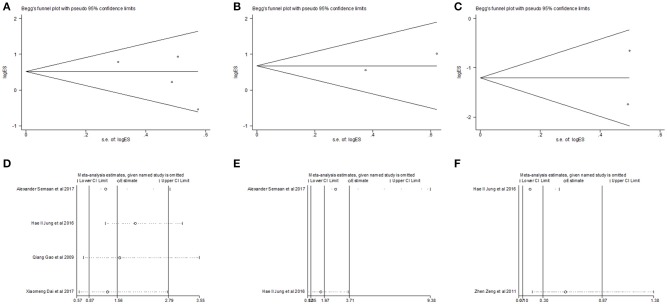
Begg's funnel plot for publication bias tests in **(A)** liver cirrhosis, **(B)** portal vein invasion and **(C)** BCLC stage. Sensitivity analysis in **(D)** liver cirrhosis, **(E)** portal vein invasion and **(F)** BCLC stage.

Begg's funnel plot and Egger's test was utilized to test potential publication bias. Sensitivity analysis was executed through sequentially omitting each trial one at a time. Figure 4 represented potential publication bias and sensitivity analysis results among studies involved in survival analysis. The consequences demonstrated that there was no apparent publication bias for OS, DFS analysis (Egger's test: *P* = 0.193 for OS, *P* = 0.806 for DFS and *P* = 0.462 for RFS). The sensitivity analysis results showed that no single trial remarkably altered the pooled results for OS, DFS, indicating that our estimates were robust and reliable. Figures S1b,c represented sensitivity analysis and potential publication bias in RFS. Besides, there was no significant publication bias for the analysis of clinical features, either (Table 2). Sensitivity analysis demonstrated that by deleting any single study, none of the pooled HRs for liver cirrhosis, portal vein invasion, and BCLC stage were remarkably affected (Figure 5). Figure S3 represented senstivity analysis and potential publication bias in remaining clinicopathological features.

## Discussion

In numerous solid tumors, PD-L1 overexpression could generate immunosuppressive tumor microenvironment and prevent T cell-mediated cytolysis, and acts as a negative regulator of T cell activation, migration, proliferation, and the secretion of cytotoxic mediators, which was accomplished by binding to PD-1 and B7.1 (CD80) (23). PD-L1 has a significant effect on preventing detrimental self-tissue destruction, which can be up-regulated on activated immune cells and directly suppress autoreactive helper T cell or play an indirect role by enabling various immune suppressive cells including T-regulatory cells (Tregs) to put a brake on autoimmunity (38–40). The immune response is responsible for the control of nascent HCC through immunosurveillance. Thus, immune checkpoint inhibitors targeting the PD-1 receptor and its ligand PD-L1, have emerged as a revolutionary treatment (41, 42). Besides, the prognostic impact of PD-L1 expression has been extensively studied in solid cancers. Previous studies have focused on the clinicopathological and prognostic significance of PD-L1 expression in patients with HCC, and clinical studies on HCC and PD-L1 continues to grow in recent years. Thus, it is essential to use an ultramodern meta-analysis for appraising the connection between the prognosis of HCC patients and PD-L1.

This meta-analysis was calculated to illuminate the association of PD-L1 over-expression with survival and clinicopathological features of HCC. Finally, 13 eligible studies containing 1,843 HCC patients were incorporated into this meta-analysis to evaluate this issue precisely. The results of the present meta-analysis suggested that PD-L1 over-expression significantly predicted positive liver cirrhosis, poorer tumor BCLC stage, positive portal invasion and poor survival. We have noticed that a meta-analysis (7) pointed out that PD-L1 expression was associated with poor DFS/PFS but not OS in patients with HCC. The difference, in conclusion, maybe because deviation existed when calculated HR from the Kaplan–Meier curves given by the articles. The differences between the included patients and clinical research on liver cancer have been ongoing could also explain it. Interestingly, one study by Sideras et al. (18) found high PD-L1 expression was connected with better overall survival, which was completely contrary to other 12 reviews. These disputed results were probably put down to the lack of specificity of anti-PD-L1 antibodies for immunohistochemical staining of PD-L1. The differences between the included patients may be another reason. Patients who underwent hepatic resection for HCC at Erasmus MC-University Medical Center (EMC, *n* = 94) or Amsterdam Medical Center (AMC, *n* = 60) were enrolled in these studies. The EMC cohort aimed to make a discovery, and AMC cohort was used to validate the results. Besides, the sample was relatively small.

In the 13 studies, Chang et al. (16) clarified that the clinical significance of PD-L1 expression in HCC patients receiving immunotherapy, it is likely that PD-L1 could serve as a marker for response to immunotherapy. However, further research is still needed because the relevant inquiry is not enough. Patients included in the remaining literature underwent surgical resection, PD-L1 expression reflected autoimmune function. Our results demonstrated that PD-L1 appeared to function as a significant biomarker in the poor prognosis of HCC and provided implications to estimate the risk of HCC patients. Patients with PD-L1 over-expression might be fit for anti- PD-L1/PD-1 from our meta-analysis. Although the significant efforts were made to conduct an integrated analysis, some shortcomings in this meta-analysis still needed to be pointed out. Firstly, the included studies were all in English, which might introduce publication bias partly though it was not found in this work. In the process of literature search in this meta-analysis, one animal study published in a language other than English was excluded so that it would have been eliminated for another reason apart from language. Secondly, since the majority of the patients in this research were from Asia, our results might not apply to other ethnic groups worldwide. Finally, the sample sizes of the included studies were too small to conduct a good pooled analysis. And not all clinicopathological features were analyzed in the meta-analysis because of the lack of related data. For example, only one study by Calderaro et al. (43) found PD-L1 expression was associated with macrovascular and microvascular invasion. Better designed clinical studies should further confirm these findings with a uniform assessment assay. Detailed clinical studies are demanded to expound the prognostic value of PD-L1 in HCC in the future.

## Conclusion

The meta-analysis demonstrated that PD-L1 high expression level forecast poorer OS and DFS in HCC patients. Furthermore, the high PD-L1 expression has relations with positive liver cirrhosis and portal vein invasion as well as the worse BCLC stage. Our results indicated that PD-L1 had great potential as a useful prognostic biomarker and a therapeutic target for HCC.

## Author contributions

W-JM conceived and designed the protocol and study. J-HL, G-GW identified studies to be screened. W-JM, J-HL, G-GW, and XJ identified studies for eligibility, extracted data and assessed the methodologic quality of included studies. XC performed the analysis with assistance from LW. X-TZ, F-LZ, Z-SL, and Y-FY revised the manuscript. All the authors read and approved the final manuscript.

### Conflict of interest statement

The authors declare that the research was conducted in the absence of any commercial or financial relationships that could be construed as a potential conflict of interest.
