# Supplementary material for: Clinicopathologic Significance and Prognostic Value of Programmed Cell Death Ligand 1 (PD-L1) in Patients With Hepatocellular Carcinoma: A Meta-Analysis
Source: Front Immunol. 2018 Sep 11;9:2077. doi: 10.3389/fimmu.2018.02077 (PMC6141709; doi:10.3389/fimmu.2018.02077)

Supporting information

Additional Supporting Information may be found in the online version of this article:

In this meta-analysis, HR and 95% CI from five studies with recurrence free survival (RFS) data demonstrated no significant association between high PD-L1 and RFS (HR=1.24, 95% CI: 0.76-2.00, P=0.39), although overt heterogeneity existed among these studies as well (Tau2=0.24; Chi^2=29.18; P<0.00001; I^2=86%) **(Figure S1a)**. Sensitivity analysis in RFS **(Figure S1b).** Begg's funnel plot for publication bias tests in RFS **(Figure S1c),**

Figure S1

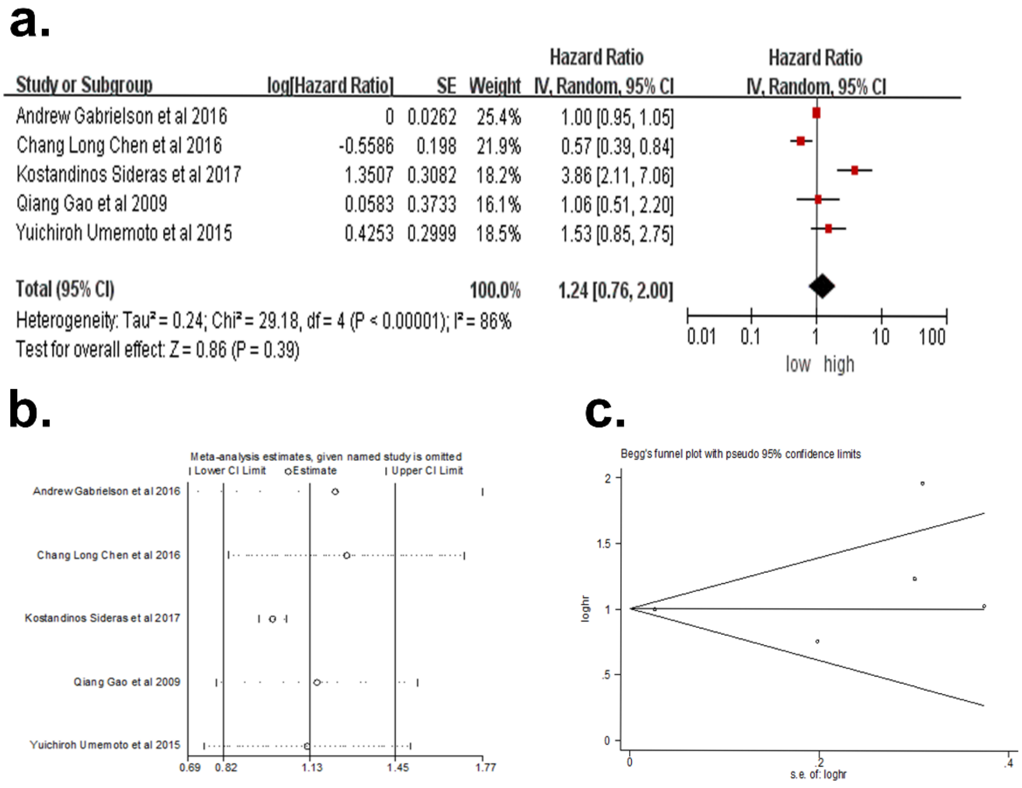

Figure S2. Forrest plot of HR for **(a)** age **(b)** sex **(c)** tumor size **(d)** AFP **(e)** Number of tumors **(f)** HBVs-Ag **(g)** An-ti HCV **(h)** vascular invasion **(i)** differentiation **(j)** TNM stage. Size of square indicates relative contribution of each study. Solid horizontal line represents 95% confidence interval of each study. Diamond indicates pooled studies.

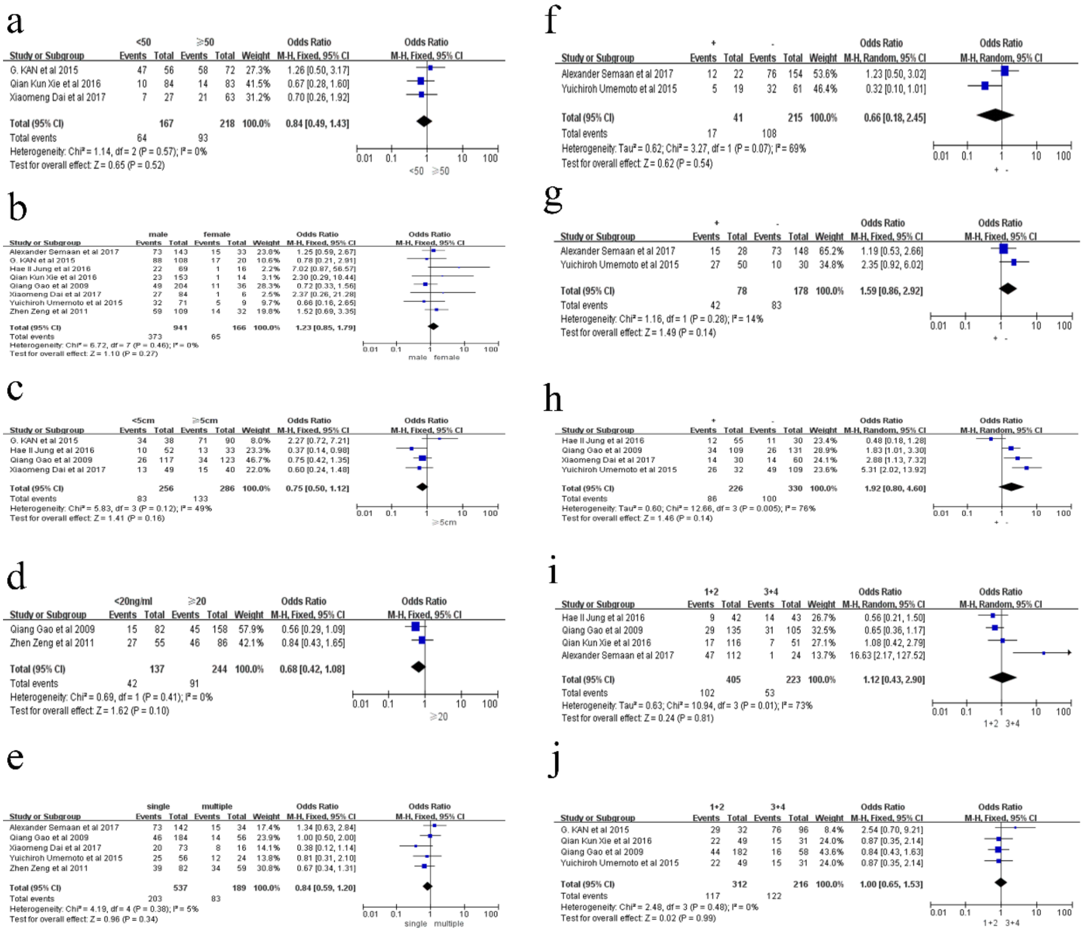

Figure S3. Begg's funnel plot for publication bias tests in **(a)** age **(b)** sex **(c)** tumor size **(d)**AFP **(e)** Number of tumors **(k)** HBVs-Ag **(l)** An-ti HCV

(m) vascular invasion **(n)** differentiation **(o)** TNM stage.

Sensitivity analysis **(f)**age **(g)**sex **(h)**tumor size **(i)** AFP **(j)** Number of tumors **(p)** HBVs-Ag **(q)** An-ti HCV **(r)** vascular invasion **(s)** differentiation **(t)** TNM stage.

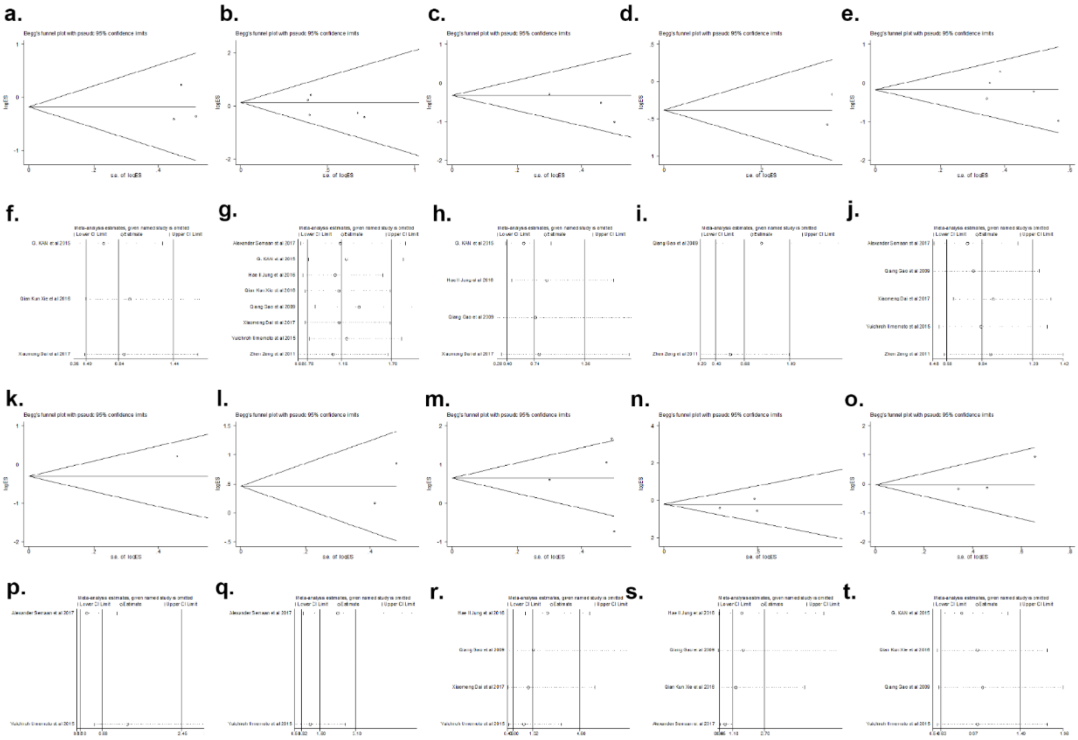

Supplement: Supplementary file 1 [file Data_Sheet_1.PDF]
